# Statistical evaluation of reader variability in assessing the diagnostic performance of optical coherence tomography

**DOI:** 10.1117/1.JBO.25.11.116002

**Published:** 2020-11-11

**Authors:** Sarah J. Erickson-Bhatt, Douglas G. Simpson, Stephen A. Boppart

**Affiliations:** a University of Illinois at Urbana-Champaign, Beckman Institute for Advanced Science and Technology, Urbana, Illinois, United States; b University of Illinois at Urbana-Champaign, Department of Statistics, Champaign, Illinois, United States; c University of Illinois at Urbana-Champaign, Department of Electrical and Computer Engineering, Urbana, Illinois, United States; d University of Illinois at Urbana-Champaign, Department of Bioengineering, Urbana, Illinois, United States; e University of Illinois at Urbana-Champaign, Carle Illinois College of Medicine, Champaign, Illinois, United States

**Keywords:** optical coherence tomography, sensitivity and specificity, diagnostic accuracy, reader variability, cancer, intraoperative

## Abstract

**Significance:** Optical coherence tomography (OCT) is widely used as a potential diagnostic tool for a variety of diseases including various types of cancer. However, sensitivity and specificity analyses of OCT in different cancers yield results varying from 11% to 100%. Hence, there is a need for more detailed statistical analysis of blinded reader studies.

**Aim:** Extensive statistical analysis is performed on results from a blinded study involving OCT of breast tumor margins to assess the impact of reader variability on sensitivity and specificity.

**Approach:** Five readers with varying levels of experience reading OCT images assessed 50 OCT images of breast tumor margins collected using an intraoperative OCT system. Statistical modeling and analysis was performed using the R language to analyze reader experience and variability.

**Results:** Statistical analysis showed that the readers’ prior experience with OCT images was directly related to the probability of the readers’ assessment agreeing with histology. Additionally, results from readers with prior experience specific to OCT in breast cancer had a higher probability of agreement with histology compared to readers with experience with OCT in other (noncancer) diseases.

**Conclusions:** The results from this study demonstrate the potential impact of reader training and experience in the assessment of sensitivity and specificity. They also demonstrate even greater potential improvement in diagnostic performance by combining results from multiple readers. These preliminary findings suggest valuable directions for further study.

## 1 Introduction

Optical coherence tomography (OCT) is emerging as a clinical diagnostic tool that is capable of providing real-time images of human tissue *in vivo* during surgical procedures.^1^^–^^15^ Wide local excision (WLE) surgery is a common treatment for solid tumors. During WLE, the goal is to remove the entire tumor and a small margin of surrounding healthy tissue without leaving any cancer cells behind.^8^ Currently, there is no real-time imaging method used as standard-of-care during WLE surgeries to assess the surgical cavity. OCT is label-free (does not require the addition or injection of dyes or probes to enhance contrast), relatively inexpensive, and capable of real-time microscopic imaging of cancer.^16^ OCT has been demonstrated as a useful tool for assessing tumor margins during surgery for human cancers such as bladder,^1^^–^^5^ cervical,^6^ breast,^7^^–^^12^ skin,^13^^,^^14^ and prostate^15^ (selected references).

In this study, we perform new statistical analyses to assess the effect of reader experience and variability on the outcome of sensitivity and specificity measures for OCT, which is used to determine the presence or absence of a positive surgical margin (tumor cells present at the cut surfaces) during WLE surgery of human breast cancers. Studies to assess the diagnostic accuracy of OCT in different organ sites varied widely in the reported literature, with sensitivity and specificity values ranging from 11% to 100%. For example, several studies were performed by Gladkova et al. to evaluate the use of OCT for detecting human bladder cancer. In each study, blinded reading of more than 100 images was performed by 7 medical doctors experienced in OCT imaging and interpretation, yielding a sensitivity of 75% to 81% and specificity of 70% to 71% for OCT, using histology as the gold standard.^1^^,^^2^ Several groups conducted studies using a commercial OCT imaging system (Niris, Imalux, Cleveland, Ohio) to image bladder cancer in patients. In these cases, the surgeon performing both the surgery and the OCT imaging also analyzed the OCT images while imaging with full knowledge of the patients’ medical records. These studies yielded sensitivities in the range of 90% to 100% and specificities in the range of 65% to 89%, when compared with the final pathological analysis.^3^^,^^5^ An OCT imaging study was performed by Escobar et al. to assess cervical cancer in patients. Blinded analysis by three clinicians who were not present during the imaging and did not have knowledge of the patients’ medical records yielded a sensitivity of 56% to 94% and specificity of 11% to 59% when compared with the final histological analysis.^6^ In a study by Assayag et al., freshly excised breast tissues were imaged using OCT and two pathologists experienced in reading OCT images performed blinded analysis, yielding a sensitivity of 90% to 94% and specificity of 75% to 79% when compared to histology.^7^ These are just a few studies that show the variability in experience and training or prior knowledge provided by readers during blinded analysis of OCT. In order to monitor the effect of reader experience on diagnostic accuracy, a study was performed by Lopater et al. where three uropathologists analyzed 119 OCT images of prostate biopsies. The readers were blinded, but after each set of 10 images, they were told the results. Readers then used this feedback and knowledge during analysis of the remainder of the images. The reader calls were then compared to final histological analysis. The average diagnostic accuracy improved by about 20% between the first and 119th images analyzed.^15^ Although many studies have been performed to assess sensitivity and specificity of OCT with various cancer types, to the best of our knowledge, this is the first study focused on extensive statistical analysis of reader variability based on prior experience.

A study previously performed by our group used OCT to assess freshly excised breast tumor margins intraoperatively to determine whether the margins were positive or negative for cancer. Blinded analysis by a single reader yielded a sensitivity and specificity of 100% and 82%, respectively.^10^ OCT was also used to image excised human lymph nodes intraoperatively. A decision tree was generated to guide three inexperienced readers in a blinded analysis, which yielded a detection sensitivity and specificity of 60% and 81%, respectively.^11^ In another study, tumor margins were imaged *in vivo* by OCT during WLE of breast cancer and a blinded reader analysis yielded an overall sensitivity and specificity of 92%.^8^ For this particular study, five readers were recruited, having varying degrees of experience in analyzing OCT images for cancer. New statistical analyses are performed here in order to analyze the impact of reader experience and variability on the performance assessment of OCT as a diagnostic imaging tool.

## 2 Methods

### 2.1 Intraoperative OCT Imaging During Wide Local Excision Surgery

A portable spectral-domain OCT system was custom built to be easily maneuvered into the operating room during surgical procedures (described in detail in Ref. 8). A custom designed handheld OCT probe was integrated into the system for imaging the surgical cavity *in vivo* following resection of the primary lumpectomy specimen. The study was performed with 35 patients undergoing surgery (22 WLE and 13 mastectomy) for breast cancer. Video-based two-dimensional (2D) cross-sectional OCT images were collected both *in vivo* from the surgical cavity and *ex vivo* from the excised tissue specimens. The excised tissue specimens were processed for routine histology and interpreted by a board-certified pathologist. The diagnoses made by the pathologist were used as the gold standard for comparison. The OCT images and videos acquired *in vivo* showed structural differences between normal and cancerous tissue within the resection bed.

### 2.2 Blinded Analysis to Assess the Sensitivity and Specificity of the OCT System

A blinded study was performed with 50 OCT images from *ex vivo* specimens (*in vivo* images were not used in the blinded study since the tissue was not resected and there was no histology for comparison). The readers were given a training set of sample OCT images showing normal adipose and stromal breast tissue as well as images portraying cancerous features. Five members of the Biophotonics Imaging Laboratory who were not involved in this study were selected to be readers based on various levels of experience. Reader 5 had no prior experience reading OCT images, readers 1 and 4 had 2 to 4 years of experience reading OCT images of noncancer types (mostly primary care, ear, nose, throat, etc.), Readers 2 and 3 had 1 to 3 years of experience reading OCT images of breast cancer (but not the images used for our study). A duplicate set of the 50 images was reversed (flipped horizontally) left-to-right and included in the test set (for a total of 100 images randomly ordered). In a single session, the readers viewed and graded all 100 images, one at a time and consecutively, without going back to an image once it was graded. Readers were not given any feedback or information about previously graded images during the session. The following grading scale (from 1 to 4) was used: a score of 1 means that the reader is confident the image is negative for cancer; a score of 2 means that the reader thinks the image is likely negative, but there is some doubt; a score of 3 means the reader thinks cancer may be present, but there is some doubt; a score of 4 means the reader is confident the image is positive for cancer. The OCT images were considered “negative” if given a score of 1 and “positive” if given a score of 2, 3, or 4. This represents the clinical scenario where additional tissue would be removed from the margin if there was “any doubt” that cancer may still be present, and a “majority vote” (3:5 readers) was used to score each image. The overall sensitivity was 91.7% (95% CI: 62.5% to 100%) and specificity was 92.1% (95% CI: 78.4% to 98%).^8^ Here, we present new and extended statistical analyses of the data from the blinded study of OCT images.

### 2.3 Statistical Analysis of Reader Experience and Variability

Statistical modeling and analysis was performed using the R language,^17^ and visualizations were created with the aid of the ggplot2 package in R.^18^ Confidence intervals for sensitivity and specificity were computed using the Wilson method,^19^ using the Hmisc library function, binconf.^20^ Receiver operating characteristic (ROC) curve analyses were performed to assess the inherent diagnostic efficacy of individual reader scores, mean scores, and median scores, the latter of which corresponds to majority voting when a call threshold is applied. ROC analyses were implemented using R libraries ROCR^21^ and auctestr.^22^ Comparing individual areas-under-the curve (AUC) with those of the combined scoring methods provides an assessment of the relative effectiveness of combined scoring methods. Reader experience levels varied, and comparison of AUC along with sensitivities and specificities across readers with different levels of training provided a means for investigating training effects.

Probabilistic image classification, using reader scores as inputs, was performed using multiple logistic regression analysis. In this analysis, the log-odds of positive histology is modeled as a linear function of the image analysis scores provided by readers or by combining readers’ scores via averaging and median. Further analysis using logistic regression provided the basis for comparing probabilistic classification performance of readers with different levels of prior training and the combination methods, compared with the gold standard of histology. To determine the statistical significance of the training effect in this experiment, a log-likelihood ratio (LLR) test compared the null logistic regression model with score effects but no training effects to a more comprehensive model with score, training as a factor main effect, and the training factor interaction with scoring included in the model.

Exploiting the two instances for each image, original and reversed, these paired scores were used to analyze the repeatability of the reader scores between the paired image presentations. Mean absolute differences between the paired scores indicated how close, on average, the repeated scores were compared to the overall variation in scores. Using the R function polychor,^23^ the polychoric correlation between the paired scores for each reader was computed to assess the level of association between scores by the same readers on the same image. Polychoric correlation treats the ordinal scores as thresholds on latent Gaussian measurements to adjust for the discreteness in the ordinal scores. Comparison of repeatability across readers with different levels of training provided another assessment of training and experience on the effectiveness of reader scoring.

## 3 Results

### 3.1 Variation in Reader Scores

**Fig. 1:**
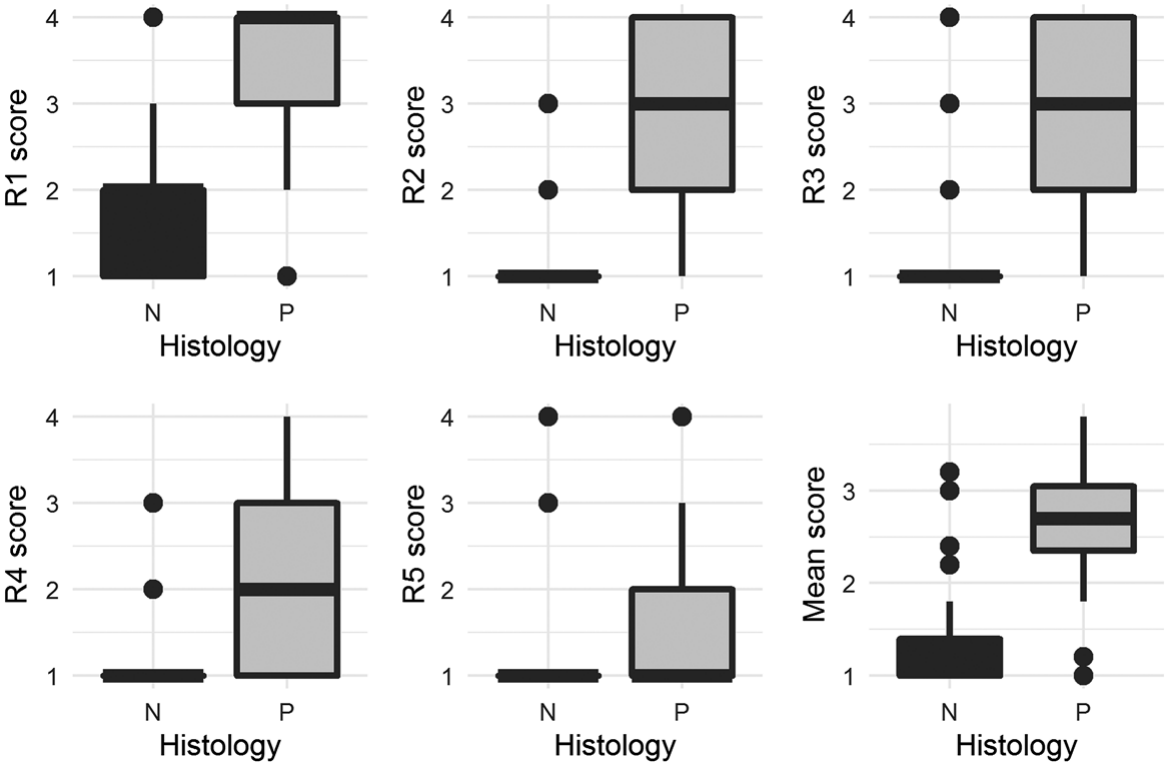
Box plots comparing distributions of individual reader scores and mean scores for positive (P) and negative (N) images. The R# labels refer to the reader number (1 to 5).

Figure 1 shows boxplots of the individual reader scores for positive and negative images. The box plots show a large median shift and no overlap for readers #2 and #3 (R2 and R3) (highly experienced readers) and R1 (moderately experienced reader), which indicates a strong scoring outcome. A smaller median shift and some overlap is shown for R4 (moderately experienced reader), which indicates a moderate scoring outcome. Complete overlap and no median shift are shown for R5 (nonexperienced reader), which indicates a poor scoring outcome. This indicates that in the population, the more experienced readers are likely to have a better scoring outcome than less experienced readers. In addition, mean scores show more precision within positive and negative images, and greater separation between positive and negative images compared to the individual reader scores, indicating likely outperformance of even the most experienced reader scoring.

### 3.2 ROC Analysis of Individual and Combined Scoring Systems

**Fig. 2:**
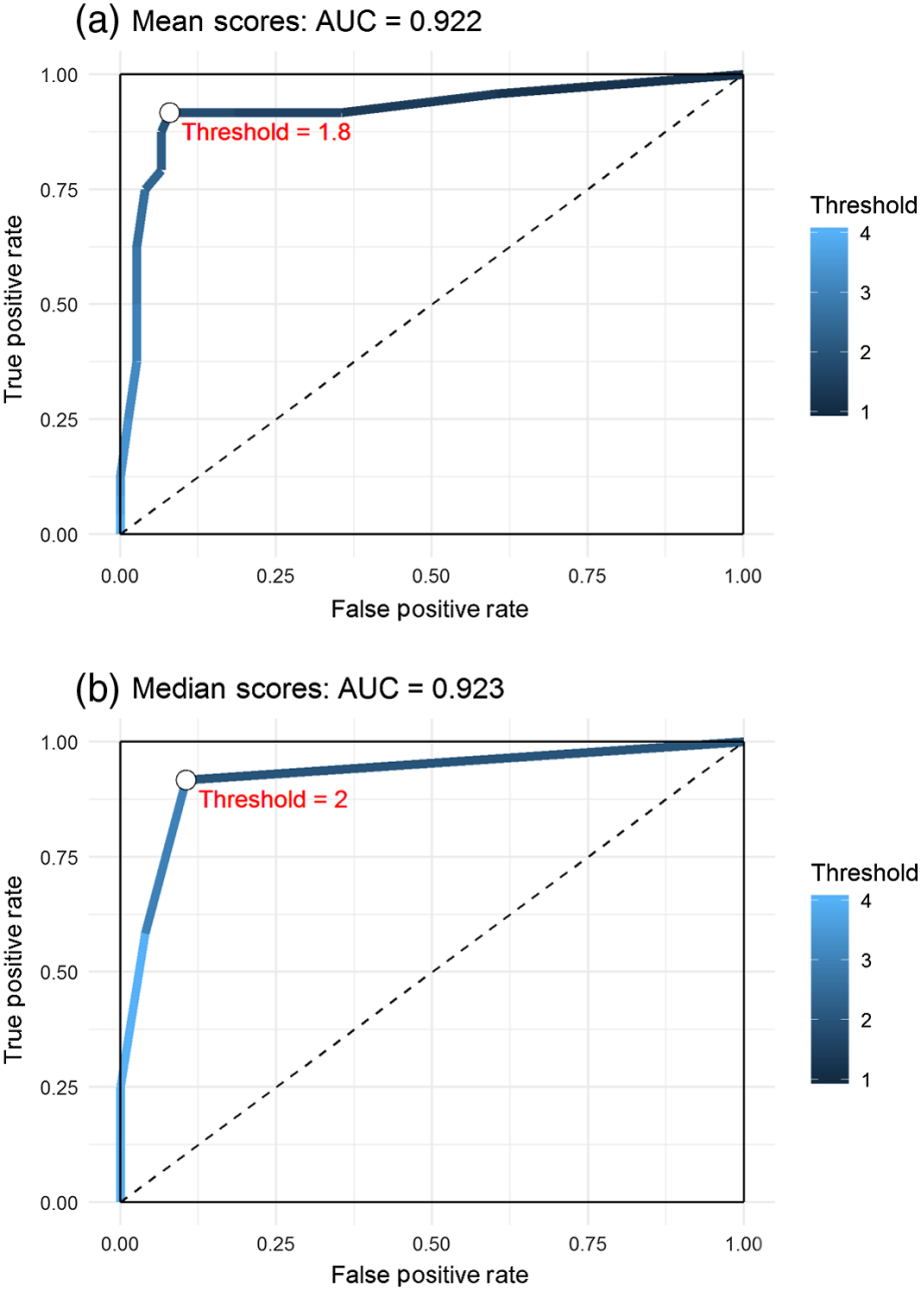
ROC curves for (a) mean score and (b) median score classification. The scale bar on the right represents the different threshold values from 1 (black) to 4 (light blue). For each, the ROC value for the optimal threshold is indicated on the curve and labeled in red.

The reader scores for each of the 100 images (50 original and 50 reversed) were combined into mean and median scores. As noted above, a threshold on median scores corresponds to majority voting for positive versus negative. Figure 2 shows the ROC curves for different thresholds on the mean score and the median score. For the mean score, a threshold of ∼1.8 is the best value for classifying the images as negative (below 1.8) or positive (above 1.8). For median scores, a threshold of 2.0 is optimal in this set, corresponding to a positive call if a majority of the five readers score 2 or higher for the image. The clinical interpretation of this threshold means that the surgeon would remove additional tissue from a margin if there was “any suspicion” that it contained cancer. Both of the combined scoring methods showed similar performances. The AUC for mean score classification was 0.922 (95% confidence interval: 0.844, 1.00), and for median score classification 0.923 (95% confidence interval: 0.846, 1.00).

**Fig. 3:**
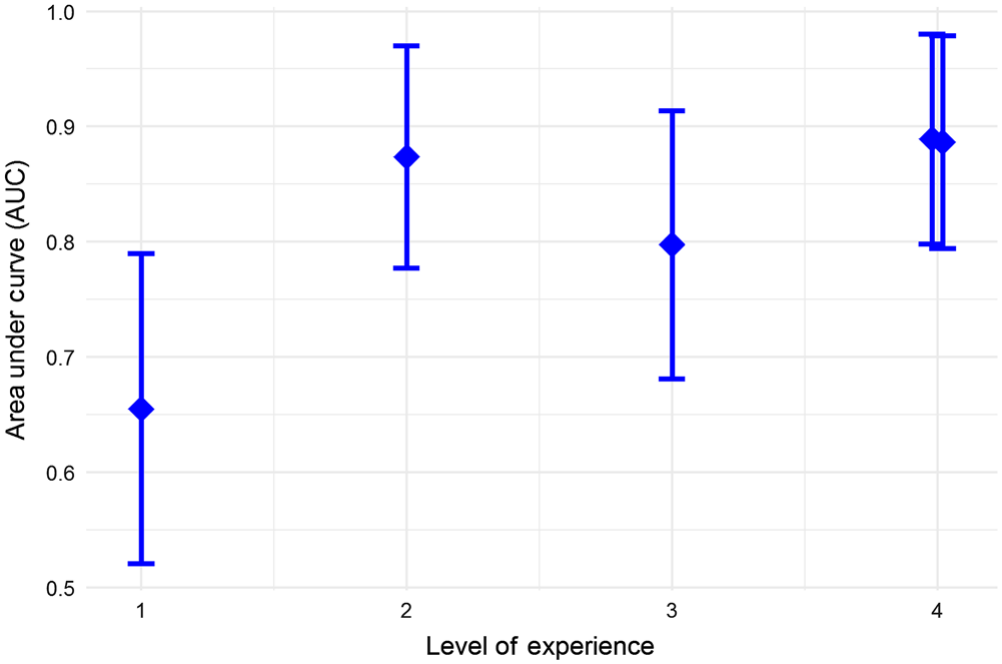
Individual reader AUC performance (±2 standard errors) versus level of experience (ordinal score from 1 to 4). The points for two readers with score=4 were offset horizontally to display their individual AUC values.

For comparison, Fig. 3 shows the AUC performance of the individual readers by levels of experience. Error bars show 95% confidence intervals computed using the equivalence of AUC to a Mann–Whitney statistic.^22^ From the individual AUC values, there is an indication of a training effect, and also an indication that the combined scoring methods outperform individual scoring, even when combining less experienced and more experienced readers.

### 3.3 Sensitivity and Specificity Analysis of Reader Variation

**Fig. 4:**
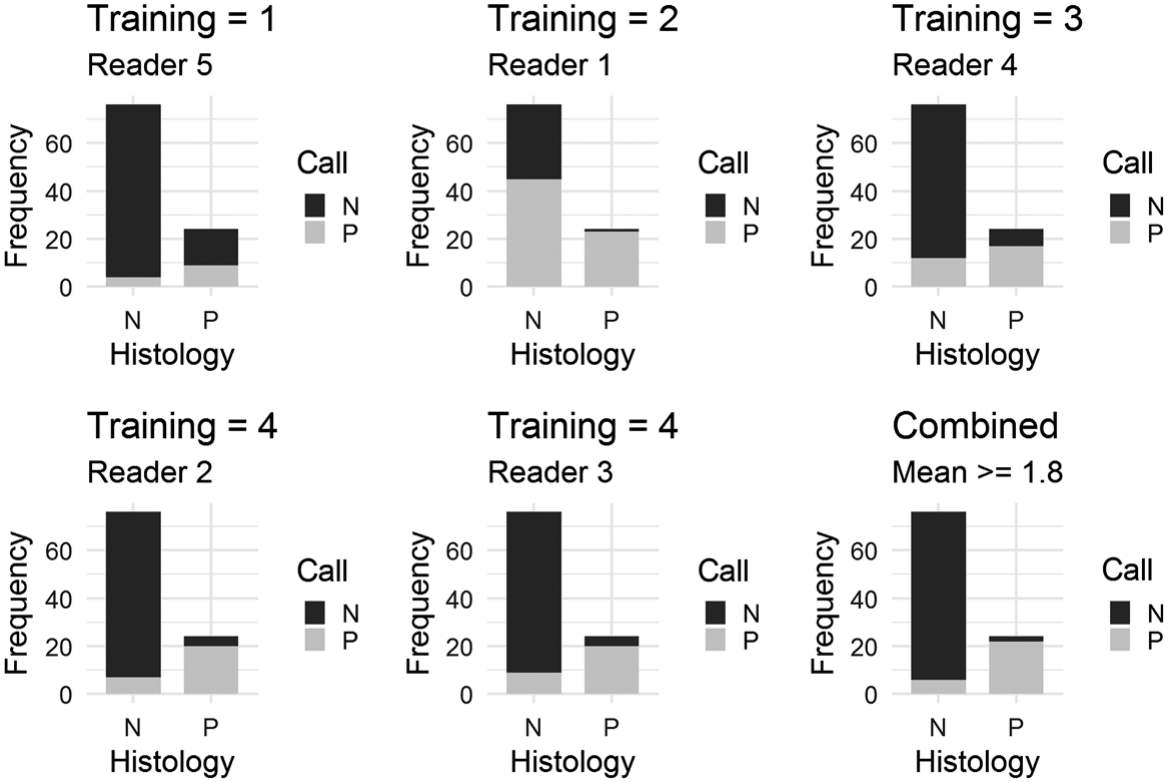
Comparison of individual reader call frequencies and mean score call frequencies versus histology (P = positive and N = negative) using threshold of 2.0 for individual reader scores and a threshold of 1.8 for mean scores; the individual reader bar plots are in increasing order of training level.

Figure 4 shows the comparison of positive and negative outcomes derived from individual reader scores versus histology. The individual reader plots are in increasing order of reader experience level. The bar plots show the number of reader calls that are positive and negative using a score threshold ≥2, for negative (left) and positive (right) images, according to histology. For a perfectly predictive set of reader scores, the left bar (histology=N) would be all dark, and the right bar (histology=P) would be all light. These plots show a general trend of increasing accuracy with an increasing level of reader experience.

For comparison, Fig. 4 also shows the frequency plots of positive and negative calls derived from mean scores using the optimal threshold of ≥1.8. This figure shows that most of the images that were negative on histology received a more negative score, on average, from the readers (below 1.8), and most of the images that were positive on histology received a more positive score, on average, by the readers (above 1.8). The results for median scores (majority≥2 versus majority<2) are nearly the same as for mean scores and are, therefore, omitted. The results indicate that images that are negative on histology are more likely to receive a negative score from readers, and images that are positive on histology are more likely to receive a positive score from readers based on the majority vote or the mean score threshold.

Combining the results summarized in Fig. 4, Table 1 provides the sensitivities and specificities for individual reader calls, majority vote calls, and mean score calls, using a threshold of 1.8. For the individual and majority vote calls, this is equivalent to a threshold of 2 due to the integer scale for individual scores. Also shown are the ordinal experience levels for the five readers in the study. This table shows a general trend toward improved sensitivity and specificity with greater experience and also indicates that the majority voting and mean scoring outperform individual readers despite combining readers with lesser and greater levels of experience.

**Table 1:** Estimated sensitivity and specificity for individual readers and combined call rules versus histology, in order of increasing reader experience (95% confidence intervals are shown in parentheses).

Experience level	Call rule	Sensitivity (95% CI)	Specificity (95% CI)
1	R5≥2	37.5 (21.2, 57.3)	94.7 (87.2, 97.9)
2	R1≥2	95.8 (79.8, 99.8)	40.8 (30.4, 52.0)
3	R4≥2	70.8 (50.8, 85.1)	84.2 (74.4, 90.7)
4	R2≥2	83.3 (64.2, 93.3)	90.8 (82.2, 95.5)
4	R3≥2	83.3 (64.2, 93.3)	88.2 (79.0, 93.6)
Mixed	Median≥2	91.7 (74.2, 97.7)	89.5 (80.6, 94.6)
Mixed	Mean≥1.8	91.7 (74.2, 97.7)	92.1 (83.8, 96.3)

**Fig. 5:**
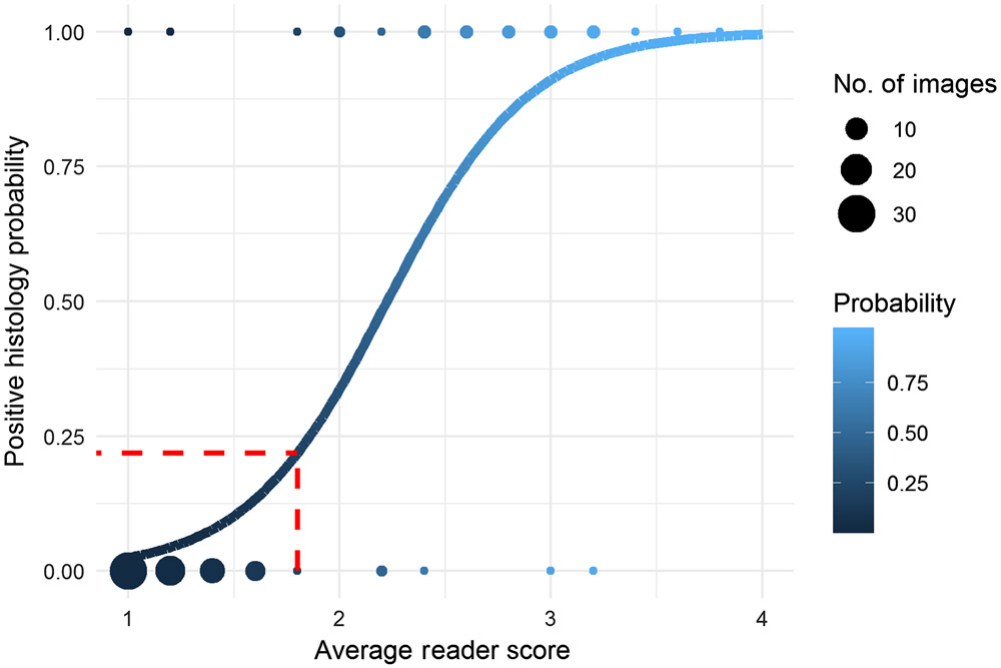
Diagnostic probability estimated from average reader scores (predictor variable) and histology calls (binary response variable) via logistic regression. The red dotted line indicates that the 1.8 threshold corresponds to a <25% likelihood of being found positive on histology.

Figure 5 shows the diagnostic probability estimated from average reader scores (predictor variable) and histology calls (binary response variable) via logistic regression. The images with average scores below 1.8 have a low probability (<25%) of being positive on histology and images with average scores above 1.8 have a higher probability (>25%) of being positive on histology. The probability of being found positive on histology increases with increasing values of average reader scores, which indicates that images scored as 2, 3, or 4 by readers are more likely to contain cancer, and the likelihood increases with higher numbers.

### 3.4 Likelihood Ratio Test of the Training Effect

It is hypothesized that greater reader experience is associated with higher classification accuracy within the probabilistic classification framework. Table 2 summarizes multiple logistic regression analysis to evaluate the association between a reader’s level of experience and the accuracy of predictive scores. Four models of decreasing complexity were fit to the log-odds of positive histology. The largest model included experience level as a factor variable along with its interactions with reader score. The simplest model had no experience related variables. The LLR chi-square test between these two models was highly statistically significant (p<0.0001), indicating that experience level is a significant variable in the accuracy of the image assessment. The best fitting model, indicated by the bold number in Table 2, and selected according to the AIC, implied a multiplicative interaction between the reader’s call score and the reader’s experience score. Thus, higher levels of experience were associated with larger score coefficients, and thus greater separation of histologically positive and negative images.

**Table 2:** Logistic (log-odds) regression analysis of reader experience as a factor in predicative accuracy of reader scores. “ExpNum” is the numerical experience score, whereas “ExpCat” is the experience level modeled as a category variable. The best model (in bold) minimizes the Akaike information criterion (AIC). LLR chi-square statistics test each model against the most complex listed first; significance of the test indicates lack of fit of the reduced model.

Model variables	AIC	LLR	Degrees of freedom	P value
Score, ExpCat, score*ExpCat	385.82	Reference	—	—
Score, ExpCat, score*ExpNum	**382.99**	1.17	2	0.556
Score, ExpCat	386.52	6.70	3	0.082
Score	406.77	32.96	6	<0.0001

**Fig. 6:**
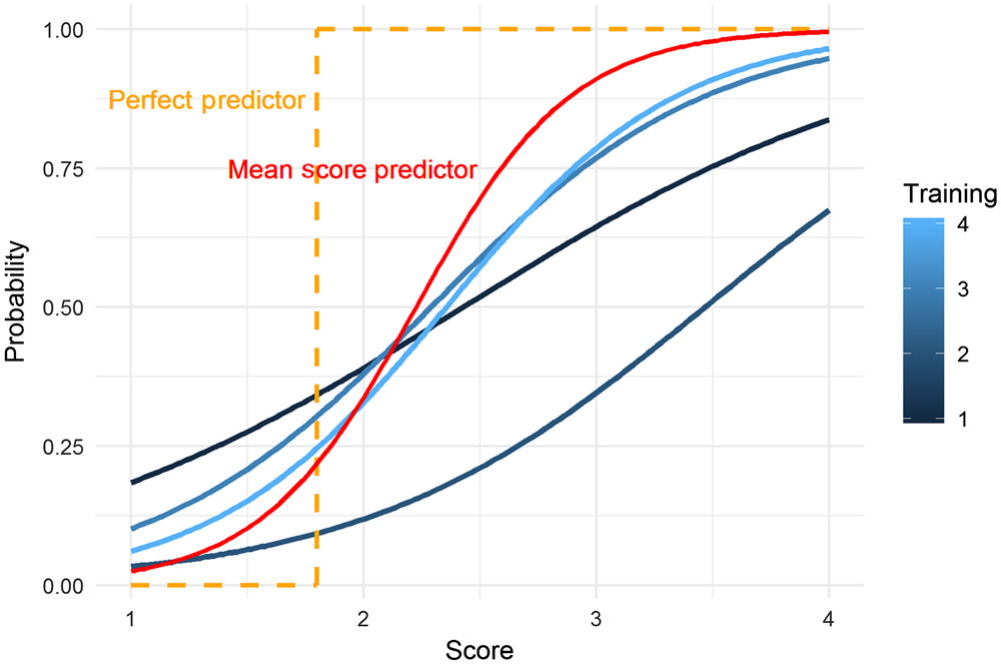
Superimposed diagnostic probability curves based on experience adjusted logistic regression analysis of histology versus reader scores and reader training levels. Fitted curves are for four levels of prior training: 1=lowest, 4=highest level of prior training. Also shown are the ideal probability curve of a hypothetical perfect predictor (orange dashed line) and the probability curve based on the mean score prediction model discussed above (red line). An additional plot of diagnostic probability curves for positive histology versus reader score is shown in the Supplementary Material.

Using the best fitting model, Fig. 6 shows the superimposed diagnostic probability curves based on experience-adjusted logistic regression analysis of histology versus reader scores and reader training levels. Fitted curves are for four levels of prior training: 1=lowest, 4=highest level of prior training. There is a statistically significant trend in the slopes of these curves (p=0.019, likelihood ratio test). The central slopes of the curves increase with the level of prior training, indicating greater separation of positive and negative histology calls by the more experienced readers. To aid in interpretation, the hypothetical probability curve of a perfect predictor is shown in the graph (orange dashed line). Better predictors are closer to this hypothetical limit. Also shown in the graph is the predictive probability curve using mean scores. The curve suggests that mean score predictions outperform the most experienced individual readers.

### 3.5 Repeatability of Image Scores versus Training

As described in Sec. 2, the five readers were presented with each image twice: the original image and the horizontally reversed mirror image. Similarity of scoring between the two presentations would indicate reproducibility of the reader’s scoring of the images. Table 3 summarizes the results of scoring original and reversed images for each of the five readers. Also included are the combined scoring methods using median (majority) and mean scorings and experience level of the reader. Two measures of reproducibility are shown: (1) the mean absolute difference between the two scores of each image for each reader and (2) the polychoric correlation between the reader’s two scores for each image. The polychoric correlation measures latent association between ordinal scores, assuming they result from thresholds on underlying Gaussian measurements.^23^

**Table 3:** Repeatability of reader scores between original and reversed images.

Experience level	Reader	Mean absolute score difference	Polychoric correlation
1	R5	0.26	0.662
2	R1	0.36	0.895
3	R4	0.20	0.916
4	R2	0.08	0.994
4	R3	0.10	0.997
Mixed	Median	0.18	0.97
Mixed	Mean	0.14	0.951

## 4 Discussion

We have shown in-depth statistical analysis of the results from a blinded reader analysis of intraoperative OCT images acquired during WLE cancer surgery. From the literature, the procedure typically followed for assessing diagnostic accuracy of OCT imaging systems involves recruiting about one to three readers to assess a test set of images and give a call of positive or negative for cancer. A subset of the total images is typically provided as a training set prior to reader analysis. The reader calls are then compared to the gold standard histology to yield the sensitivity and specificity. Our previous study by Erickson-Bhatt et al.^8^ differed in that five readers were recruited with varied previous experience reading OCT images and the scoring was based on a scale rather than a binary positive or negative call. The scale gives readers more flexibility in assessing images for cancer, and the ROC analysis (Fig. 2) showed that the best threshold is 1.8 or 2 for the mean and median scores, respectively, which corresponds to a positive call for scores of 2 or higher and resulted in greater accuracy corresponding to histology. If the readers were only given the option of binary positive/negative calls then many of the scores of “2” might have been incorrectly called as negative. The threshold also better represents the actual clinical setting where the surgeon would remove additional tissue from the margin if there was “any suspicion” that it was cancerous.

The AUC performance of the individual readers by levels of experience (Fig. 3) showed that reader accuracy improved with increased level of previous experience. A likelihood ratio test derived from multiple logistic regression confirmed this conclusion (p<0.0001). This might appear to be an expected result as more “training” leads to greater reader accuracy; however, in this case, the previous reader experience was from images using different OCT systems and even different (noncancer) applications, but still resulted in improved reader accuracy. This is useful information because the number of images in training sets can be limited because they must be removed from the overall image data set. Our statistical analysis shows that including additional OCT images from other systems and studies to supplement the reader training will improve reader accuracy. We also found the unexpected result that the combined scores (mean and median of the five readers) resulted in greater sensitivity and specificity than any individual scores (from even the most experienced readers) despite combining readers with lesser and greater levels of experience (Figs. 4 and 6). Furthermore, combined scores exhibited a high degree of repeatability, nearly as high as scores of the most experienced readers (Table 3).

Based on our experience with this study, it was beneficial to provide a reasonable training set to the readers to enhance their familiarity with the variation in images, both positive and negative. We also found it useful to augment the images by providing reverse images to increase the number of tumor margin images for training. Indeed, readers with greater prior experience reading OCT images performed better individually in our small study. Therefore, it can reasonably be suggested to augment reader training with additional images from historical data if available and if needed to provide them with sufficient training.

Other studies have shown agreement that experience and training are important for reader success. A study by Trindade et al.^24^ showed that volumetric laser endoscopy had high accuracy among experienced users. Another study by Wessels et al.^25^ showed that three inexperienced students could achieve high accuracy when trained by experts.

Using a scoring scale from 1 to 4 for the reader evaluations of the images, we were able to compare how different readers would have higher or lower optimal binary thresholds based on ROC analysis. For example, Fig. 4 demonstrates that setting a binary call threshold at score 2 or greater leads to different call results for different readers, and potentially loses information that was recovered from their four level scores. This refinement allows readers to show some doubt or suspicion, while still offering the opportunity for a binary call threshold if desired.

We also found it valuable to recruit multiple readers, both to be able to compare inter-reader variation, and to allow for combined scores across multiple readers (five readers) to improve diagnostic performance as we found in our study.

Once OCT systems reach a clinical setting, the surgeons or physicians who use them will be well-trained to make accurate diagnoses. In the meantime, for experimental studies to assess initial diagnostic accuracy, we believe these recommendations will lead to improved and systematic blinded reader analysis. We also expect that these guidelines would translate to the improvement in blinded reader analysis of other imaging methods beyond OCT.

## 5 Conclusion

Extensive statistical analysis was performed on reader variability in OCT image analysis. The analyzed results were from a blinded analysis by five readers of OCT images acquired intraoperatively from breast tumor margins. Sensitivity and specificity values based on scores from individual readers varied greatly and corresponded to three levels of previous experience analyzing OCT images. This statistical study demonstrates the potential impact of experience and adequate training of readers when assessing the diagnostic accuracy of OCT imaging in clinical studies. Within the scope of our study, reader experience was shown to be a statistically significant factor in reader scoring classification accuracy and reproducibility. A surprising preliminary finding was that mean score and majority voting methods based on combining information from individual reader scores improved the accuracy of classification, even compared to the most experienced readers. We believe that this use of multiple readers is a promising direction for future studies and one that bears further study.
