# Supplementary material for: Statistical evaluation of reader variability in assessing the diagnostic performance of optical coherence tomography
Source: J Biomed Opt. 2020 Nov 11;25(11):116002. doi: 10.1117/1.JBO.25.11.116002 (PMC7657413; doi:10.1117/1.JBO.25.11.116002)
Supplement: Supplementary file 1 [file JBO_025_116002_SD001.pdf]

Supplemental Material

Figure S1 shows the diagnostic probability curves estimated from individual reader scores (predictor variable) and histology calls (binary response variable) via logistic regression. The individual curves show the estimated probability of having a positive histology outcome for a given OCT image as a function of the reader score for the image. The curves were estimated by logistic regression, with the histology code (1 or 0) as the response and the reader score as the predictor variable. The curves from Readers #1, 2, and 3 (Figure 6 C-E) show high accuracy in scores of 1 and 4, corresponding to negative or positive results, respectively, on histology, and lower accuracy in scores of 2 and 3. The curve from Reader #4 (Figure 6D) shows high accuracy in a score of 1 and lower accuracy for scores of 2, 3, and 4. The curve from Reader #5 (Figure 6E) shows lower accuracy for all scores when compared to the average. The pink curve was generated using the average scores from the 5 readers. The curves from R2 and R3 (highly experienced readers) and R4 (moderately experienced reader) show that the scores have greater probability of corresponding to histology outcomes while the curve from Reader #5 (unexperienced reader) shows that the scores have a much lower probability of corresponding to histology outcomes. The curve from R1 (moderately experienced reader) shows that lower scores have a high probability of being found negative on histology, but the higher scores do not have such a high probability of being found positive on histology. This is due to a high number of false positives in the scoring from R1.

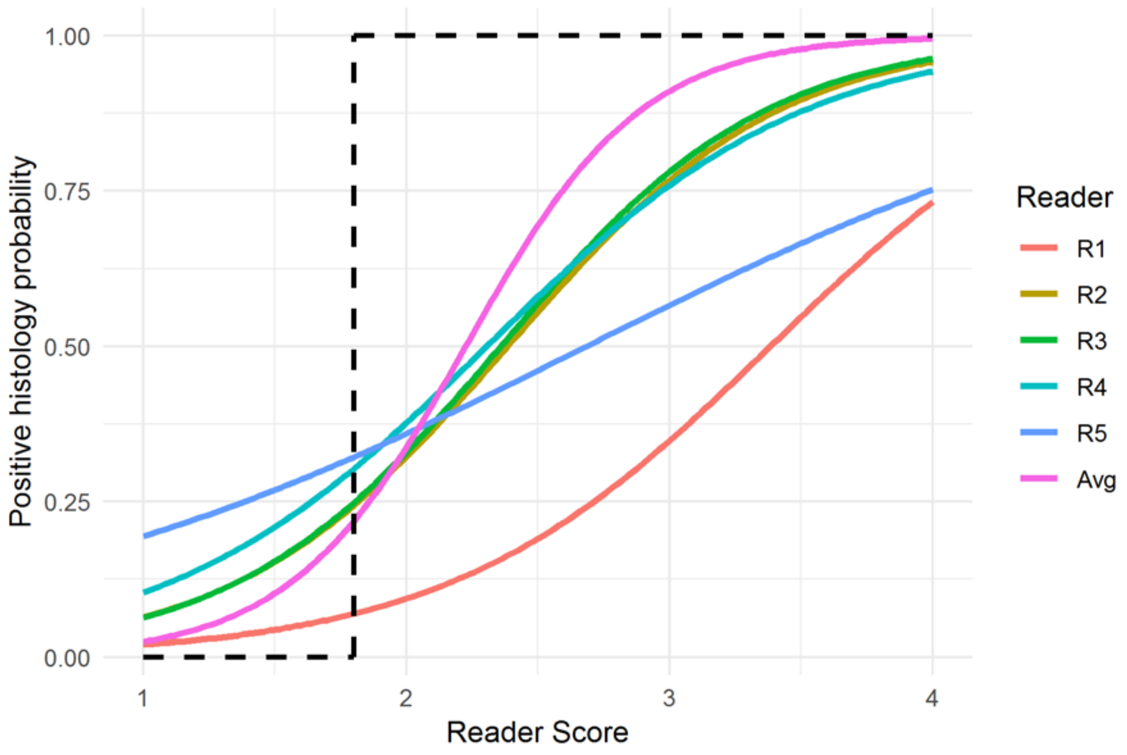

Fig. S1 Superimposed diagnostic probability curves for positive histology versus reader score; the predictive curve for the average reader score (pink) is steeper than the individual reader curves, indicting increased discriminatory power compared with individual reader scores. Dashed line indicates the hypothetical probability curve for a perfect predictor.
